# Draft Genome Sequences of *Mycoplasma alkalescens*, *Mycoplasma arginini*, and *Mycoplasma bovigenitalium*, Three Species with Equivocal Pathogenic Status for Cattle

**DOI:** 10.1128/genomeA.00348-13

**Published:** 2013-06-13

**Authors:** Lucía Manso-Silván, Florence Tardy, Eric Baranowski, Aurélien Barré, Alain Blanchard, Marc Breton, Carole Couture, Christine Citti, Emilie Dordet-Frisoni, Virginie Dupuy, Patrice Gaurivaud, Daniel Jacob, Claire Lemaitre, Macha Nikolski, Laurent-Xavier Nouvel, François Poumarat, Patricia Thébault, Sébastien Theil, François Thiaucourt, Pascal Sirand-Pugnet

**Affiliations:** CIRAD, UMR CMAEE, Montpellier, Francea; INRA, UMR1309 CMAEE, Montpellier, Franceb; Anses, Laboratoire de Lyon, UMR Mycoplasmoses des Ruminants, Lyon, Francec; Université de Lyon, VetAgro Sup, UMR Mycoplasmoses des Ruminants, Marcy L’Etoile, Franced; INRA, UMR 1225, IHAP, Toulouse, Francee; Université de Toulouse, INP, ENVT, UMR 1225, IHAP, Toulouse, Francef; Université de Bordeaux, Centre de Bioinformatique et Génomique Fonctionnelle, Bordeaux, CBiB, Bordeaux, Franceg; INRA, UMR 1332 de Biologie du Fruit et Pathologie, Villenave d’Ornon, Franceh; Université de Bordeaux, UMR 1332 de Biologie du Fruit et Pathologie, Villenave d’Ornon, Francei; Université Bordeaux, LaBRI, UMR 5800, Talence, Francej

## Abstract

We report here the draft genome sequences of *Mycoplasma alkalescens*, *Mycoplasma arginini*, and *Mycoplasma bovigenitalium*. These three species are regularly isolated from bovine clinical specimens, although their role in disease is unclear.

## GENOME ANNOUNCEMENT

*Mycoplasma alkalescens*, *Mycoplasma arginini*, and *Mycoplasma bovigenitalium* are bacteria of the class *Mollicutes* clustered within the hominis phylogenetic group. They have been associated with disease in cattle, but their contribution to pathogenesis remains unclear. *M. alkalescens*, described in 1973 (1), has been reported from mastitis in cattle and from arthritis, otitis, and pneumonia in calves (2–5). In pneumonic calves, *M. alkalescens* is often associated with *Mycoplasma bovis*, but recent recurrent isolation in the United Kingdom in the absence of other pathogens suggests it may constitute an emerging mycoplasma (6). *M. arginini*, characterized in 1968 (7), is a much more ubiquitous species isolated from a broad collection of mammalian hosts (8). It has been associated with various pathologies in ruminants and is often found in association with *M. bovis* in cattle (3). However, its pathogenicity has never been established (9), and this species is best known as a frequent contaminant of eukaryotic cell cultures (10). *M. bovigenitalium*, characterized in 1955 (11), comprises the strains of the *Mycoplasma* ovine/caprine serogroup 11, reassigned in 2008 (12). It has been associated with reproductive disorders in ruminants (2, 13, 14) and has proven to induce pneumonia in gnotobiotic calves (9).

Genome sequences of the most relevant mycoplasmal bovine pathogens (*Mycoplasma mycoides* subsp. *mycoides* and *M. bovis*) have already been published. To widen the availability of genome data from mycoplasmas of bovine origin, we present here the genome sequences of *M. alkalescens*, *M. arginini*, and *M. bovigenitalium*.

Selected strains were isolated in France from lung tissue samples from calves with pneumonia. *M. alkalescens* strain 14918 and *M. arginini* strain 7264 were isolated in 2007, the latter being found in association with *Mannheimia haemolytica*. *M. bovigenitalium* strain 51080 was isolated in 2009 from a septicemic calf, again concomitantly with *M. haemolytica*. Whole-genome sequences were obtained using a combination of Illumina (single reads) and 454 (mate paired with 8-kb insert size). Assembly was performed using Newbler 2.3, and annotation was conducted using a customized version of the CAAT-Box platform (15), with automatic preannotation for coding sequences followed by expert validation, as detailed previously (16). Genome analysis and comparisons were mainly conducted using the MolliGen 3.0 platform (17).

The general properties of the three genomes are shown in Table 1. Sequences related to integrative conjugative elements were found in both *M. alkalescens* and *M. bovigenitalium*, whereas a prophage, similar to that previously described in the small ruminant pathogen *Mycoplasma agalactiae* (18), was identified in *M. bovigenitalium*. These mobile genetic elements constitute an important driving force of genome plasticity and may be associated with horizontal gene transfer among *Mycoplasma* species sharing the same habitat (16, 19).

**TABLE 1:** General properties of the three genome sequences

Characteristic	*M. alkalescens* 14918	*M. arginini* 7264	*M. bovigenitalium* 51080
No. of contigs >500 bp	20	18	42
Median coverage	126×	158×	44×
GenBank accession no.	AMWK00000000	AORG00000000	AORH00000000
Genome size (bp)	771,939	615,621	862,247
G+C (%)	25.56	26.22	28.96
Gene density (%)	85.18	90.24	88.5
No. of CDSs*^a^*	601	513	677
No. of pseudogenes	33	9	24
No. of structural RNA genes	37	36	36

a CDSs, coding sequences.

Comparative genome analysis of mycoplasmas of bovine origin displaying diverse pathogenicity, as well as host and tissue tropism, will improve our understanding of the evolution of bovine mycoplasmas and will pave the way for unraveling the genetic basis of mycoplasma pathogenicity and host specificity.

### Nucleotide sequence accession numbers.

Draft genome sequences of *M. alkalescens*, *M. arginini*, and *M. bovigenitalium* were deposited as Whole-Genome Shotgun projects at GenBank under the accession no. AMWK00000000, AORG00000000, and AORH00000000, respectively.
